# Effects of an integrative warm-up method on the range of motion, core stability, and quality of squat performance of young adults

**DOI:** 10.3389/fspor.2024.1323515

**Published:** 2024-03-27

**Authors:** Tijana Šćepanović, Miloš Kojić, Mladen Mikić, Valdemar Štajer, Uğur Ödek, Ana Penjak

**Affiliations:** ^1^ Faculty of Sports and Physical Education, University of Novi Sad, Novi Sad, Serbia; ^2^ Faculty of Sport Sciences, Bektaş Veli Üniversitesi, Bektas, Turkey; ^3^ Faculty of Kinesiology, University of Split, Split, Croatia

**Keywords:** integrated method, self-myofascial release, warm-up, squat, young adults

## Abstract

**Introduction:**

This research aims to determine the effects of an integrative warm-up method on the range of motion in joints of the lower extremities, the strength of the stabilizer trunk muscles, and the quality of the basic movement patterns in older adolescents.

**Methods:**

The study sample consisted of 88 male students (age 20.1 ± 0.5). They were randomly divided into four groups: one control group (CG) (*n* = 17; 180.8 ± 7.9 cm; 82.3 ± 8.3 kg) and three experimental groups (EG): EG1 (*n* = 23; 180.9 ± 7.0 cm; 78.5 ± 9.5 kg), EG2 (*n* = 31; 182.2 cm ± 7.3 cm; 79.5 ± 11.5 kg), and EG3 (*n* = 17; 183.3 ± 4.9 cm; 77.5 ± 11.8 kg). The participants were subjected to a 6-week experimental treatment: EG1 once, EG2 twice, and EG3 three times a week. The experimental treatment consisted of four sub-phases representing the integrative warm-up Method: 1) Inhibition (self-myofascial release using a foam roller); 2) Lengthening (Static stretching in a maximum range of motion position); 3) Activation (Positional isometrics muscle activation of the trunk and gluteus); 4) Integration (Integrated all the previous phases into one complex movement pattern). Based on the covariance analysis (ANCOVA), statistically significant treatment effects were observed and positive changes were determined in all experimental groups.

**Results:**

The differences between groups were observed in the following variables: Overhead Squat Assessment (*p* = 0.000; $\;\eta_{p}^{2}=0.318$), range of motion of left hip flexion (*p* = 0.000; $\eta_{p}^{2}=0.371$), range of motion of right hip flexion (*p* = 0.000; $\eta_{p}^{2}=0.051$) and range of motion of right hip extension (*p* = 0.051; $\eta_{p}^{2}=0.088$), Double Leg Lowering Test (*F* = 2.411; *p* = 0.014; $\eta_{p}^{2}=0.014$) and range of combined motion (plantar and dorsiflexion) of left ankle joint (*p* = 0.000; $\eta_{p}^{2}=0.299$). There was no significant difference in the Plank Test (*F* = 1.007; *p* = 1.007; $\eta_{p}^{2}=0.035$), range of combined motion (plantar and dorsiflexion) of right ankle joint (*p* = 0.088; $\eta_{p}^{2}=0.170$) and range of motion of left hip extension (*p* = 0.158; $\eta_{p}^{2}=0.060$). The participants of CG statistically significantly differed from EG1, EG2, and EG3 in the squat performance after the applied treatment.

**Discussion:**

The effect of the treatment was the occurrence of a transformational processes in almost all measured variables. It can be concluded that the integrative method is effective and applicable in practice for both young adults and recreational athletes.

## 1 Introduction

Integrative Method (IM) is a way of training that aims to improve every component needed to perform movements at the highest level, as well as prevent injury (1). IM is a very interesting and practical method because it can include static stretching exercises, activation of muscles, and integration of isolated exercises and movements into one complex movement pattern. Designing this exercise program is completely different from any other type of exercise in which a person or athlete engages. It is similar to stretching and relaxation training, however, to be successfully implemented, observing how a person reacts to an exercise is essential. In addition, it can include self-myofascial release (SMR) under the guidance of a trained therapist or a personal trainer (1).

Foam roller self-massage, better known as SMR, is an integral part of IM. The myofascial system is a protective 3D network matrix of connective tissue. It envelops all muscles and surrounds the nervous and musculoskeletal system (2). SMR works not only on muscles and tendons, but it can also loosen soft tissue adhesions and scar tissue, which can provide benefits similar to those of stretching or massage. Foam rolling is an easily applied technique to relieve tension and acutely improve joint range of motion without accompanying deficits in muscle performance (3).

Self-myofascial release with foam rolling has been widely used over the last decade. The purpose of SMR is to improve the flexibility of soft tissue and to reduce pain (4). Many studies have shown that SMR can increase the range of motion (ROM) of the hip, knee, and ankle joints, without impairing muscle strength (3, 5, 6). SMR is a common technique used by many athletes and patients to help in recovery, improve ROM, and prevent injuries (7–12). When performing SMR exercises and using foam roller as a tool, it is important to pay attention to the force with which we press the muscle against the roller in order not to lose the appropriate line of execution of the exercise. Otherwise, we will not get the desired effect from myofascial release.

The IM is already an established training approach with a positive transfer to many aspects of the body (1). The idea of this research arises from the desire to explore whether the IM, in the form of a warm-up, can also have positive effects on the parameters mentioned in the title. This approach to studying the IM as a warm-up opens up possibilities for a better understanding of the impact of this method on specific research goals, including increasing range of motion, core stability, and improving fundamental movements. It is important to note that the Integrative warm-up method takes the same amount of time as the usual warm-up procedure.

Core stability is an important component of postural and functional status, which ensures a static and dynamic balance of the body. IM can be used as a corrective exercise method for imbalances in the human body (13). An integrated postural exercise program might lead to a more balanced muscle efficiency, inducing positive changes in athletes (14). Injuries and chronic imbalances in the muscles might occur due to minimal imbalances that preceded and were not noticed on time. Such conditions require a serious approach to treatment and long-term therapy (15). In addition to the fact that certain imbalances in the muscles are not noticed on time, certain segments of exercise, such as strengthening and stretching of certain muscle groups, are completely neglected. This additionally affects the possibility of injuries that often occur in athletes, as well as in the rest of the population. For this reason, scientists suggest that corrective exercise can positively affect the prevention and recovery from injuries and chronic muscle imbalances (10, 16).

The other important component of functional movement performance is flexibility. Previous research has shown that improving ROM in joints has a positive impact on the overall health status (17, 18). Functional ROM is defined as the minimum range of motion necessary to perform daily activities comfortably and to live efficiently (19–23). The change and increase of ROM in the joints can be influenced by static stretching exercises, but due to the acute negative effects of static stretching on muscle strength, interest in SMR has been steadily increasing (24). Many studies have shown that SMR can increase hip, knee, and ankle ROM without impairing muscle strength (3, 5, 6). That is why it is crucial to include daily training and activities with integrated movements and exercises. These contribute to improved ROM, better posture, and, consequently, a healthier overall condition. Given the effects that occur with the use of this type of corrective exercise, IM is widely used in rehabilitation, sports, and recreation (1). The most appropriate screening method for assessing movement quality is the method according to the National Academy of Sports Medicine (1, 25).

The Squat, as a natural functional movement of the body, is a strength exercise at the same time. Athletes use it to improve their performance and, in the case of clinical patients, to improve their health in everyday activities (26). In clinical settings, the squat strengthens lower-body muscles and connective tissue after joint-related injury (27). That is why, in this paper, the quality of squat performance is the subject of research.

Despite the proven importance of IM, the authors noted that there is little available research on the effect of IM as a form of warm-up. This research aims to determine the effects of an IM warm-up on increasing ROM in lower limb joints, strengthening weakened trunk stabilizer muscles, and improving basic movement patterns. The above-mentioned review of the literature considers that IM contains several components that can have positive effects on ROM, core stability, and quality of squat performance. Thus, hypothesis H1 is defined by which we expect differences between control and experimental groups of respondents who use IM warm-up once, twice, or three times a week.

## 2 Materials and methods

### 2.1 Study design

This research is experimental, lasting 6 weeks with 4 groups, of which 3 are experimental and one is a control group. IM treatment was administered to the experimental groups. The control group was not subjected to treatment and these individuals spent their time as usual. All respondents were healthy people and had not reported any health problems. The testing was performed by a professionally trained person at the Kinesitherapy lab, Faculty of Sports and Physical Education in the morning hours. The subjects were instructed not to engage in intense physical activity 24 h before the test, and to eat and sleep normally.

### 2.2 Participants

The sample consisted of a total of 88 male students (age 20.1 ± 0.5 years; height: 181.8 cm ± 6.9 cm; weight: 79.4 kg ± 10.4 kg) who participated in the research. The minimum sample size (*n* = 74) was determined through power analysis utilizing G*Power software (Heinrich-Heine-Universität Düsseldorf). The effect size was set at 0.50 (considered a medium effect size), with an alpha error probability of 0.05, a power of 0.80 for four groups, and two measurements of study outcomes. The respondents filled out a survey according to which they were classified as moderately to highly physically active, an average of 10 h a week. The study was of open-label type for students at University, and all participation was voluntary and they could leave the study whenever they wanted. The participants were systematically randomly divided into four groups (selecting every third respondent): one control and three experimental groups. The first group, the control group (CG) (*n* = 17), was not subjected to IM. Experimental E1 (*n* = 23), E2 (*n* = 31), and E3 (*n* = 17) groups were subjected to a 6-week IM (E1 once, E2 twice, and E3 three times a week).

### 2.3 Data collection

All subjects were initially tested before the start of IM and after 6 weeks at the final test. Basic anthropometric measurements were taken: body height and weight. Anthropometric variables were measured following the norms of the International Biological Program (28). Body height was measured using Martin's anthropometry (GPM Anthropometer 100; DKSH Switzerland Ltd., Zurich, Switzerland; ±0.1 cm) and body mass was measured with a digital scale (BC1000, Tanita, Tokyo, Japan; ±0.1 kg).

#### 2.3.1 Range of motion assessment

ROM was measured with a goniometer in the hip joint and ankle joint. Based on the measurement of flexion and extension in hip joints and collective mobility in the ankle joints (plantar and dorsiflexion), the following variables were obtained: hip flexion, hip extension and range of motion in ankle joints. Both legs were tested individually.

#### 2.3.2 Strength assessment

For assessment of strength, endurance and stability of trunk, the Plank Test was used (29). The result is expressed in seconds. Participants are required to assume the plank position, holding a static position engaging the muscles of the trunk and the entire body for as long as possible. The test concludes when participants break the proper isometric contraction position or discontinue due to muscle fatigue. Another core stability test is the Double Leg Lowering Test (DLL). It is used to assess abdominal muscle performance, which has been proven to be reliable in research (30). Testing is performed from the supine position, raising both legs. During the test, the lumbar part of the back is attached to the ground so that the pelvic tilt is posterior. During DLL the pelvis should remain still. The protractor is placed on the wall with marked angles of 90°, 75°, 60°, 45°, 30° and 15°. The result is measured at the moment when the participant loses control over the pelvis. When the degree is lower, the result is better.

The quality of the squat performance was tested by the Overhead Squat Assessment (1). This test is recorded with a camera and the result is subsequently processed and analyzed. The participant should perform 5 squats in the frontal plane with the face to the camera, 5 squats in the sagittal plane and 5 squats in the frontal plane but with the back to the camera. Certain parameters are observed in each of the 3 positions relative to the camera. In the frontal plane, while the participant is facing the camera, the position of the knees and feet (rotated outward or flat) is observed. In the sagittal plane, the lumbo-pelvic hip complex (LPHC) is evaluated. Deviations from normal are excessive arching, hyperextension in the lumbar spine, or forward flexion of the trunk. In the frontal plane with the back facing the camera, the symmetry of the pelvis (movement to the right or left) is assessed. Also, the position of the pelvis, Achilles tendon, and feet are observed. Each of the above-mentioned body segments should be in the correct, physiological position during the movement. Each segment is evaluated individually. If any of the body segments deviates from the normal position, it is considered a deviation and is assigned a score of (1), while a score of (0) is assigned if the segment remains in the normal physiological position. The sum of all the marks gives the final mark for the quality of the performance of the movement pattern. The values of this test are inverse, which means that a higher score indicates a worse result on the test, and a lower score characterizes a better result.

#### 2.3.3 Experimental treatment

The IM follows a model of warm-up exercise that confirms four segments according to the “NASM Essentials of Corrective Exercise Training” (1). It consists of SMR with a roller for 30 s for every muscle region, passive stretching of the same muscle groups for 30 s, activation and strengthening of muscles, and integration of exercises into a complex movement. The total duration of one treatment is 15 min and includes warming up for training (Table 1).

**Table 1:** Experimental treatment.

Training phase	Exercise	Sets	Duration, reps	Notes
Inhibition	Self-myofascial release			Maximum pressure on the roller
	Gastrocnemius soleus	1	30 s	
	Hamstrings	1	30 s	
	Adductors	1	30 s	
Lengthening	Static stretch			Relaxed breathing
	Gastrocnemius	1	30 s	
	Soleus	1	30 s	
	Supine hamstring	1	30 s	
	Kneeling hip flexor	1	30 s	
Activation	Isolated strengthening			Opposite side abductors
	Hip bridge	1	10	
	Ball crunch	1	60 s	
	Side plank with hip abduction	1	10	
	Plank	1	60 s	
Integration	Ball wall squat with overhead press	1–2	15	

The experimental treatment consisted of four sub-phases representing the integrative warm-up method: (1) Inhibition (self-myofascial release using a foam roller); (2) Lengthening (Static stretching in a maximum range of motion position); (3) Activation (Positional isometrics muscle activation of the trunk and gluteus); (4) Integration (Integrated all the previous phases into one complex movement pattern). In the first phase of treatment, participants treated all muscle groups from the back of the leg, posteriorly SMR for 30 s (1. m. soleus, m. gastrocnemius, Achilles tendon, 2. m. semitendinosus, m. semimembranosus, m. biceps femoris, 3. m. gluteus maximus, m. gluteus medius, m. gluteus minimus, 4. m. adductor longus, m. adductor brevis, m. adductor magnus). First, all the muscles on the right, and then the same on the left leg. The subjects were asked to exert maximum pressure on the roller, with their entire body weight.

In the second phase of IM method, the participants statically stretched their muscles for 30 s in a maximum range of motion position. In the position of standing step, muscle groups from the back of the lower leg were stretched. After that, in the lying position on the back, a group of hamstrings muscles were stretched. At the end of the second phase, the participants stretched the hip flexors statically in a kneeling position.

The third phase was related to the activation of the muscles of the trunk and gluteus. The participants alternately performed exercises to activate the trunk and buttocks muscles for 30 s on both sides. The order of activation of muscle groups was performed following by the model in Table 1. The isolated strengthening of muscle groups involved the first activation of the hip extensor muscles, using the Hip Bridge exercise. The next exercise was the Ball Crunch for strengthening Global Core Stabilizers, then Side Plank with Abduction and, at the end, Plank.

In the fourth phase of IM, the participants tried to integrate all the previous phases of the exercise into one, through a complex movement pattern. The essence of the whole treatment is to improve the basic movement pattern and to eliminate unwanted movements. Proper movement, ROM in the joints, body posture, and overall health are that way ensured and preserved.

There was no rest between exercises, and each exercise was performed with body weight.

### 2.4 Statistic

The data were analyzed using the SPSS (version 20.0, IBM Corp., Armonk, NY, USA). A test of normality, the Kolmogorov–Smirnov test (KS) was used to determine the distribution of data, which were found to be normally distributed. In addition, Levene's test was applied to check the homogeneity of variance. The descriptive statistics (M ± SD) for the pre- and post-tests were calculated for all groups. We looked at the time-by-group interaction effects to see whether estimated changes over time were dependent on the participants' group (twelve separate 2 × 4 mixed-design ANOVA for each performance measure). After that, we calculated the mean differences between the initial and final testing measurements for each group on *p* ≤ 0.05 and *p* ≤ 0.01 level of significance [95% confidence intervals]. Analysis of covariance (ANCOVA) was used to compare the effects of treatments between groups so that the variables from the initial one were the covariables at the final measurement. Between groups, *post hoc* pair-wise comparisons were performed using the LSD procedure.

## 3 Results

Estimated mean changes (mean difference from initial to final testing [95% CIs) within the groups from the 2 × 4 ANCOVA models are presented in Table 2. Results indicate significant interaction (group × time) in Overhead Squat Assessment (*p* = 0.000; $\eta_{p}^{2}=0.318$), range of motion of left hip flexion (*p* = 0.000; $\eta_{p}^{2}=0.371$), range of motion of right hip flexion (*p* = 0.000; $\eta_{p}^{2}=0.051$) and range of motion of right hip extension (*p* = 0.051; $\eta_{p}^{2}=0.088$), Double Leg Lowering Test (*F* = 2.411; *p* = 0.014; $\eta_{p}^{2}=0.014$) and range of combined motion (plantar and dorsiflexion) of left ankle joint (*p* = 0.000; $\;\eta_{p}^{2}=0.299$).

**Table 2:** General linear models (a 2 × 4 mixed design ANOVA) and standardized mean differences [95% confidence intervals] from initial to final testing.

Variable		Pre-test	Post-test	Mean change [95% CIs]	df	2 × 4 mixed ANOVA: group-by-time interaction effect		
Group		Mean ± SD	Mean ± SD			*F*	*P*	$\eta_{p}^{2}$
Plank (s)	CG	170.35 ± 92.45	170.88 ± 106.36	−0.53 (−70.15, 69.09)	16	1.007	1.007	0.035
	EG1	152.08 ± 37.41	163.82 ± 77.21	−65.03 (−101.09, 28.97)	22			
	EG2	169.61 ± 93.14	193.06 ± 93.54	−23.45 (−70.88, 23.98)*	30			
	EG3	182.52 ± 62.96	217.11 ± 70.89	−34.59 (−81.43, 12.25)**	16			
DLL (°)^a^	CG	29.11 ± 12.40	26.47 ± 11.28	2.65 (−5.64, 10.93)	16	3.743	0.014	0.014
	EG1	31.95 ± 10.41	28.04 ± 9.38	0.54 (−5.35, 6.44)**	22			
	EG2	41.61 ± 14.34	34.35 ± 15.58	7.26 (−0.35, 14.87)**	30			
	EG3	32.35 ± 19.61	31.41 ± 20.40	0.94 (−13.04, 14.92)	16			
Squat^a^	CG	2.65 ± 1.73	2.65 ± 1.73	1.00 (−1.21, 1.21)	16	13.037	0.000	0.318
	EG1	2.91 ± 1.67	2.04 ± 1.52	0.87 (−0.25, 1.99)**	22			
	EG2	2.55 ± 1.43	1.13 ± 1.14	1.42 (0.52, 2.32)**	30			
	EG3	2.29 ± 1.96	0.41 ± 0.71	1.88 (0.85, 2.91)**	16			
Right leg hip flexion (°)	CG	75.52 ± 8.76	72.35 ± 11.46	−0.47 (−6.53, 5.59)	16	11.592	0.000	0.051
	EG1	73.17 ± 6.02	76.00 ± 8.56	−7.59 (−11.02, −4.16)**	22			
	EG2	70.41 ± 8.35	79.32 ± 6.41	−8.90 (−12.69, −5.12)**	30			
	EG3	72.35 ± 11.46	80.76 ± 8.99	−8.41 (−15.61, −1.21)**	16			
Left leg hip flexion (°)	CG	74.94 ± 10.28	75.82 ± 9.58	−0.88 (−7.83, 6.06)	16	16.532	0.000	0.371
	EG1	73.04 ± 8.12	77.30 ± 7.05	−6.96 (−11.48, −2.43)**	22			
	EG2	68.74 ± 7.62	78.19 ± 7.54	−9.45 (−13.31, −5.60)**	30			
	EG3	72.47 ± 9.38	80.00 ± 8.15	−7.53 (−13.67, −1.39)**	16			
Right leg hip extension (°)	CG	29.17 ± 3.98	28.17 ± 4.69	0.999 (−2.04, 4.04)	16	2.691	0.051	0.088
	EG1	27.04 ± 4.64	29.17 ± 4.54	−2.19 (−4.92, 0.54)**	22			
	EG2	27.80 ± 5.19	28.87 ± 4.39	−1.06 (−3.51, 1.38)*	30			
	EG3	29.00 ± 5.76	29.23 ± 3.89	−0.24 (−3.67, 3.20)	16			
Left leg hip extension (°)	CG	28.58 ± 3.98	28.35 ± 4.04	0.24 (−2.57, 3.04)	16	1.776	0.158	0.060
	EG1	25.69 ± 5.01	27.86 ± 3.94	−3.30 (−5.99, −0.62)**	22			
	EG2	27.83 ± 4.89	29.38 ± 4.55	−1.55 (−3.95, 0.85)**	30			
	EG3	27.29 ± 5.58	29.00 ± 4.09	−1.71 (−5.13, 1.72)	16			
Right ankle joint (°)	CG	71.94 ± 5.76	73.00 ± 8.07	−1.06 (−5.96, 3.84)	16	5.724	0.088	0.170
	EG1	72.91 ± 7.62	74.91 ± 7.40	−6.56 (−11.02, −2.09)*	22			
	EG2	71.80 ± 8.55	76.35 ± 8.45	−4.55 (−8.87, −0.23)**	30			
	EG3	71.94 ± 8.43	79.47 ± 10.01	−7.53 (−14.00, −1.06)**	16			
Left ankle joint (°)	CG	72.76 ± 6.34	72.17 ± 6.18	0.59 (−3.79, 4.97)	16	11.961	0.000	0.299
	EG1	72.17 ± 7.45	74.52 ± 8.45	−6.71 (−11.45 −1.97)**	22			
	EG2	70.93 ± 8.22	76.80 ± 8.53	−5.87 (−10.13, −1.61)**	30			
	EG3	71.29 ± 8.09	78.88 ± 10.99	−7.59 (−14.34, −0.84)**	16			

CG, control group; EG (1, 2, 3), experimental groups.

^a^ Variable with opposite metric orientation; df - degrees of freedom; *F* – statistics; *p* – significant difference of *p* ≤ 0.05; $\eta_{p}^{2}$ - Partial Eta squared.

* Significant difference at *p* ≤ 0.05.

** Significant difference at *p* < 0.001.

On average, participants in EG1 significantly improved DLL (*p* < 0.01), Overhead Squat Assessment (*p* ≤ 0.01), Right leg hip flexion (*p* ≤ 0.01), Left leg hip flexion (*p* ≤ 0.01), Right leg hip extension (*p* ≤ 0.01), Left leg hip extension (*p* ≤ 0.01), Right ankle joint (*p* ≤ 0.05), Left ankle joint (*p* ≤ 0,01); EG2 improve all measures and EG3 improve Plank (*p* ≤ 0.01), Overhead Squat Assessment (*p* ≤ 0.01), Right leg hip flexion (*p* ≤ 0.01), Left leg hip flexion (*p* ≤ 0.01), Right ankle joint (*p* ≤ 0.05), Left ankle joint (*p* ≤ 0,01). Moreover, control group did not improve any measures.

Further analysis of the difference between the means and significance of pairwise comparisons revealed statistically significant differences between the groups of participants, thus confirming the hypothesis about the influence of IM. The participants of CG are statistically significantly different from EG1, EG2, and EG3 in the quality of performing squats, right leg hip flexion and extension, and left ankle joint. The quality of the basic movement pattern, which was tested through the squat, was best performed by EG3 (0.41 ± 0.71), then EG2 (1.13 ± 1.14), then EG1 (2.04 ± 1.52); the control group had the lowest grade (2.65 ± 1.7). As far as ROM was concerned, the groups differed statistically significantly from each other, gradually from the lowest results at the final measurement of the control group, to the best results in the experimental group with three treatments per week. Table 3 presents detailed information on the results of the post-hoc analysis.

**Table 3:** Results of the post-hoc analysis.

Variable group		CG	EG1	EG2	EG3
Plank (s)	CG	–	0.641	0.193	0.193
	EG1	0.641	–	0.361	0.184
	EG2	0.193	0.361	–	0.589
	EG3	0.193	0.184	0.589	–
DLL (°)^a^	CG	–	0.586	0.134	0.338
	EG1	0.586	–	0.296	0.122
	EG2	0.134	0.296	–	0.010*
	EG3	0.338	0.122	0.010*	–
Squat^a^	CG	–	0.002*	0.000**	0.000**
	EG1	0.002*	–	0.003*	0.000**
	EG2	0.000**	0.003*	–	0.023*
	EG3	0.000**	0.000**	0.023*	–
Right leg hip flexion (°)	CG	–	0.001*	0.000**	0.000**
	EG1	0.001*	–	0.094	0.223
	EG2	0.000**	0.094	–	0.797
	EG3	0.000**	0.223	0.797	–
Left leg hip flexion (°)	CG	–	0.006**	0.000**	0.000**
	EG1	0.006*	–	0.001*	0.065
	EG2	0.000**	0.001*	–	0.204
	EG3	0.000**	0.065	0.204	–
Right leg hip extension (°)	CG	–	0.004*	0.019*	0.205
	EG1	0.004*	–	0.414	0.120
	EG2	0.019*	0.414	–	0.357
	EG3	0.205	0.120	0.357	–
Left leg hip extension (°)	CG	–	0.005*	0.060	0.081
	EG1	0.005*	–	0.213	0.341
	EG2	0.060	0.213	–	0.890
	EG3	0.081	0.341	0.890	–
Right ankle joint (°)	CG	–	0.557	0.038*	0.003*
	EG1	0.557	–	0.110	0.007*
	EG2	0.038*	0.110	–	0.146
	EG3	0.003*	0.007*	0.146	–
Left ankle joint (°)	CG	–	0.049*	0.000**	0.000**
	EG1	0.049*	–	0.022*	0.003*
	EG2	0.000**	0.022*	–	0.252
	EG3	0.000**	0.003*	0.252	–

CG, control group; EG (1, 2, 3), experimental groups.

^a^ Variable with opposite metric orientation.

* Significant difference at *p* ≤ 0.05.

** Significant difference at *p* < 0.001.

## 4 Discussion

The aim of the research was to examine the effects of a 6-week modern integrated warm-up method on the range of motion in the joints of the lower extremities, strengthening the trunk stabilizer muscles, and improving basic movement patterns in older adolescents. There is currently very little evidence of the effect of IM warm-ups on movement functionality. IM caused changes in the mobility of the joints and the quality of the squat.

By looking at the results and in comparison with the previous research, it can be concluded that some authors obtained similar results on the effectiveness of SMR application (3, 5, 6, 24). However, it must also be emphasized that the volume of the previous research differs from this research. Previous studies were based on rolling that lasted longer, while in this research rolling lasted 30s, in the form of warming up for the upcoming training. In fact, there is no consensus on how long the foam-rolling sequence should last (31). Moreover, the mechanism by which the effect is achieved is still unclear and insufficiently researched (32). Future research should determine the specific physiological and biomechanical mechanism on the changes in the muscle structure by applying this treatment.

It is interesting to note that the effects of IM were also noted in the EG1 group, although they practiced IM once a week for 6 weeks. In fact, they only had 6 treatments. EG1 is statistically significantly different from the control group in 6 out of 9 tested variables. The ROM of flexion of the hip, knee, and ankle joints is important for the quality of the squat performance. The obtained results can be compared with the research in which the application of SMR plantar facials managed to increase the posterior muscular chain flexibility (33). The authors explain this phenomenon by the fact that the plantar fascia is the most distal part of the muscle chain, and that applying pressure on it stimulates mechanoreceptors that enable relaxation of the entire chain (31). After six weeks, there were large changes in the increase in the range of motion of the ankle, particularly in EG3. The results themselves follow the logic of the very choice of exercises in the IM exercise program to increase mobility (10). The exercises focused on relaxing the ligaments and muscles in order to increase mobility. The effectiveness of the applied treatment has not been proven on core stability. Similar results were obtained in the previous study, where the group of participants who used SMR did not experience significantly positive effects on the core (10).

Based on the results of our study, it is evident that the second experimental group (EG2), subjected to treatment twice a week, demonstrated the most significant positive impact compared to the control group and the other experimental groups. This finding suggests that the frequency of treatment application plays a crucial role in achieving optimal results. EG2, undergoing treatment twice a week, exhibited a substantial improvement compared to the other groups. This reinforces the assumption that proper dosage and distribution of the treatment play a key role in attaining the desired effects. The presented results support the idea that further research should be directed towards investigating the optimal time intervals between treatments to achieve the best outcome.

The core is activated before large body movements as part of the postural control system (34, 35). Core stability affects the effective use of the required strength and endurance (36), which results in the correct body position when performing squats with arms overhead. During the squat test, there is a flexion of the hip joint, which partially straightens the lumbar spine. In this way, squat depth depends more on hip flexibility than trunk stability. This could be the reason why the results indicate that IM had a greater effect on joint mobility than on core stabilizer strength and endurance.

The effects of IM on the quality of performing the basic movement pattern of the squat are as expected. The last phase of the IM concept is precisely the integration of the previous phases of the exercise into one the quality of squat performance. The group applying IM, 3 times per week achieved the best results of all four groups, followed by the group applying it 2 times per week, and then by the group doing it 1 time per week. The participants of the control group, who had not undergone the experimental treatment, received the lowest scores. Thus, the hypothesis has been confirmed.

Some authors got the opposite results. For example, the effects of a similar treatment, as a warm-up method, on squat performance measured by EMG, did not show a better performance in contrast to the dynamic warm-up method in the previous research (37). At this point, it is relevant to highlight that future research tests the effects of IM heating treatment on other basic movement patterns and looks for transformational changes in them.

There are basic limitations of this study. The subjects in this study are healthy and physically active students who tend to engage in sports and could not reduce physical activity for the study. In the following research, the quality of squat performance with arms overhead, shoulder mobility, but also the strength/endurance of trunk extensor muscles, and the influence of these muscle groups on the effects analyzed in this research should be considered. On the other hand, the positivity and strength of this research lies in the fact that it is very repeatable and allows for the necessary changes to be made in the direction of more regressive or progressive exercises according to the available clinical conditions and subjects.

## 5 Conclusions

The integrated warm-up method is a good way to prepare the musculoskeletal system for subsequent training. By using myofascial massage in the first phase, the muscles and ligaments are relaxed, and in the second and third phases, the muscles are activated by static stretching and strengthening with simple exercises. The research results have provided us with information that even with minimal exercise of 1 time per week, it is possible to obtain the effects of the exercise. It can be said that IM can be practical and applicable in practice for both young adults and recreational athletes.

## Data Availability

The original contributions presented in the study are included in the article/Supplementary Material, further inquiries can be directed to the corresponding author.
